# Tumor-related IGF2BP1-derived molecular subtypes to predict prognosis and immune microenvironment in head and neck squamous cell carcinoma

**DOI:** 10.3389/fimmu.2024.1469435

**Published:** 2024-10-24

**Authors:** Qin Ding, Mingzhu Liu, Yuhui Pan, Ziyi Wu, Jing Wang, Yi Li, Xiaoyong Liu, Jinghua Lai, Dan Hu, Sufang Qiu

**Affiliations:** ^1^ Department of Radiation Oncology, Clinical Oncology School of Fujian Medical University, Fujian Cancer Hospital (Fujian Branch of Fudan University Shanghai Cancer Center), Fuzhou, China; ^2^ Fujian Provincial Key Laboratory of Translational Cancer Medicine, Fuzhou, China; ^3^ Department of Pathology, Clinical Oncology School of Fujian Medical University, Fujian Cancer Hospital (Fujian Branch of Fudan University Shanghai Cancer Center), Fuzhou, China

**Keywords:** tumor microenvironment, IGF2BP1, molecular subtypes, head and neck squamous carcinoma, RNA modification

## Abstract

**Background:**

Recent studies have underscored the biological significance of RNA modifications in tumorigenicity and progression. However, the potential roles of RNA modifications in immune regulation and the formation of the tumor microenvironment (TME) in head and neck squamous carcinoma (HNSC) remain unclear.

**Methods:**

We collected 199 untreated HNSC samples and clinicopathological data from Fujian Provincial Cancer Hospital. MeRIP-seq and RNA-seq were performed to generate methylation and gene expression profiles, respectively. Consensus molecular subtyping was employed to identify prognosis-related genes and RNA modification patterns in HNSC. Experiments confirmed the potential oncogenic behavior influenced by key genes. Molecular subtypes were identified through consensus clustering and validated using external cohort validation sets.

**Results:**

Among the RNA modification-related genes, IGF2BP1 emerged as the most prognostic. HNSC patients were categorized into high and low IGF2BP1 expression groups. High-expressing patients exhibited poorer survival and reduced chemosensitivity, coupled with increased tumor mutational burden, low PD-L1 expression, and limited immune cell infiltration, indicative of aggressive disease. Analysis revealed two distinct RNA modification patterns associated with IGF2BP1 expression: biosynthetically intense type (BIT) and oncogenically active type (OAT), each characterized by distinct clinical features, outcomes, and biological pathways. In an independent immunotherapy cohort, BIT patients displayed enhanced immune responses and sustained clinical benefits.

**Conclusions:**

This study highlights the crucial link between RNA modification and TME diversity. Evaluating RNA modification in tumors improves our understanding of TME features and supports the development of effective immunotherapy strategies.

## Introduction

RNA modification, involving chemical group addition to RNA nucleotides, governs RNA functions (1). Modifications like m1A, m6A, m6Am, m5C, m7G, ac4C, m3C, and Ψ regulate gene expression, affecting mRNA stability, splicing, translation, and localization (2, 3). RNA modification related genes (RMGs), including writers, erasers, and readers, orchestrate these processes, crucial for cellular function (1, 4). Dysregulation of RMGs may lead to aberrant cell growth and survival, particularly in cancer (5). RMGs impact tumor development by disrupting gene expression, presenting potential therapeutic targets (6, 7). Understanding RNA modification mechanisms is pivotal for comprehending cancer pathogenesis and devising novel therapeutic strategies.

Head and neck squamous cell carcinoma (HNSC) is a highly lethal malignancy with significant mortality rates despite treatment advancements (8–10). Extensive studies on mRNAs, lncRNAs, and EVs have identified numerous biomarkers and therapeutic targets; however, precise prognostic markers remain critically lacking (11–14). Genetic aberrations drive HNSC pathogenesis, influencing tumor initiation, progression, and therapy resistance (15, 16). Research has unveiled mutations in oncogenes and tumor suppressor genes, disrupting signaling pathways and fostering uncontrolled cell growth (17, 18). Yet, the comprehensive genetic landscape, notably in RNA modification regulators, and their impact on HNSC progression remain incompletely elucidated. Bridging this gap is imperative for tailored therapeutics and enhanced patient outcomes in HNSC.

This study endeavored to (i) characterize genetic variations and identify prognostic RMGs, (ii) investigate their functional roles in HNSC biology and treatment responses, and (iii) delineate novel molecular subtypes to refine HNSC classification and assess clinicopathological features.

## Methods and materials

### Cell culture

SCC7 and CAL27 cell lines were obtained from the Fujian Cancer Hospital Cell Bank and cultured under optimal conditions to ensure robust growth and viability. The cells were grown in Dulbecco’s Modified Eagle Medium (DMEM), which was supplemented with 10% fetal bovine serum (FBS). 1% penicillin-streptomycin was included in the medium. The cells were maintained in an incubator set to 37°C with a humidified atmosphere of 5% CO_2_, closely mimicking physiological conditions. Transfections used siNC and siIGF2BP1 RNA sequences (19, 20).

### Collection of clinical samples

This study included two cohorts. The first cohort comprised 3 cases of HNSC tumor tissue and 3 cases of normal tissue for meRIP-seq analysis. The second cohort was utilized for RNA-seq analysis. Fresh tumor biopsy specimens were obtained from 193 head and neck cancer patients at Fujian Cancer Hospital (January 2015 - January 2018, **Table 1**). Tumor classification and staging followed the TNM system. The research was approved by the Ethics Committee of Fujian Cancer Hospital (Fujian Branch of Fudan University Shanghai Cancer Center, approval number K2022-084-01). The written consent of all participants was obtained in advance. External validation data from The Cancer Genome Atlas (TCGA) included 504 tumor samples and 44 normal samples.

### Total RNA isolation, construction, and sequencing of mRNA library

Total RNA was extracted from the tumor tissues using the TRIzol reagent kit, following the manufacturer protocol to ensure optimal yield and purity. To enrich the mRNA, oligo(dT)-attached magnetic beads were employed, selectively binding to the poly(A) tails of mRNA molecules. The enriched mRNA was then fragmented using the Optimal Dual-mode mRNA Library Prep Kit (BGI-Shenzhen, China) to facilitate subsequent cDNA synthesis. Reverse transcription of the fragmented mRNA into complementary DNA (cDNA) was performed, creating a double-stranded cDNA library. In addition to repairing the ends, these purified double-stranded cDNA fragments had their 3’ ends modified by adding an adenine nucleotide. This A-tailing step is crucial for the subsequent adapter ligation. Adapters, containing sequences necessary for amplification and sequencing, were ligated to the cDNA. The adapter-ligated cDNA was then amplified through PCR to ensure sufficient quantities for sequencing. Afterwards, BGI Technology Services Co. Ltd. sequenced the cDNA library, utilizing advanced sequencing platforms to generate high-quality data for further analysis. Detailed experimental procedures are available in the **Supplementary Materials**.

### MeRIP-seq and bioinformatic analysis

Total RNA was isolated and fragmented into ~100 nt pieces using a fragmentation buffer. The RNA was split into two parts: one for input and the other enriched with an m6A-specific antibody. Enriched RNA was transcribed into cDNA using random primers, end-repaired, and ligated to Illumina adaptors, creating a library sequenced on an Illumina NovaSeqTM 6000. Fastp (v0.20.0) filtered the sequencing data to obtain high-quality reads by eliminating adaptor-containing, high-N content, poly-A, and low-quality reads. ExomePeak2 (v1.0.0) was used for peak calling, identifying read-enriched regions (p < 0.05) as peaks. Peak-associated genes were validated using genomic position and gene annotation data. Peak distribution in 3’UTR, 5’UTR, and CDS regions was assessed. MEME suite and DREME were used for motif analysis in peak-associated transcript sequences.

### Transwell assay for cell migration and invasion

For the invasion assay, transwell inserts were coated with 50 µL of Matrigel diluted 1:8 in serum-free medium (SFM) and incubated at 37°C for 30 minutes to solidify. After solidification, 1 × 10^5^ cells suspended in 200 µL of SFM were seeded into the upper chamber of each insert. The lower chamber was filled with 600 µL of complete medium containing 10% fetal bovine serum (FBS) as a chemoattractant.

For the migration assay, Matrigel was not applied, but all other procedures remained consistent. Cells were incubated at 37°C in a humidified atmosphere with 5% CO_2_ for 24 hours for migration and 48 hours for invasion assays. After incubation, non-migratory cells on the upper surface of the membrane were removed with a cotton swab. Migratory cells on the lower surface were fixed with 4% paraformaldehyde for 15 minutes, stained with 0.1% crystal violet for 10 minutes, and washed with PBS. Cells were counted under a microscope in three randomly selected fields per insert.

### Cell counting kit 8 assay

After seeding 1000 cells per well in 96-well plates for uniform growth, overnight incubation was followed by addition of 10 μL CCK8 reagent to each well to evaluate cell viability and proliferation via colorimetric changes. Cells were then cultured for specified durations (3h, 6h, 9h, and 12h), and absorbance was measured at 450 nm using a multifunctional microplate reader at these intervals. This methodology provides a quantitative assessment of cell proliferation and viability over time.

### 5-ethynyl-2-deoxyuridine assay

EdU cell proliferation assays were conducted using the BeyoClick™ EdU Cell Proliferation Assay Kit. All subsequent procedures were conducted according to the manufacturer instructions. For each experimental group, cells were subjected to treatment with EdU at a concentration of 10 μmol/L for 2 hours. Fluorescence microscopy was employed for visualizing and recording fluorescent signals.

### Single-cell data acquisition and processing

Single-cell RNA sequencing data were obtained from the Gene Expression Omnibus (GEO) database under the accession number GSE103322. For data processing, we employed the Seurat R package to perform quality control, normalization, and scaling of the single-cell data. We conducted dimensionality reduction using principal component analysis (PCA) and Uniform Manifold Approximation and Projection (UMAP) to visualize the data in lower-dimensional space. Subsequently, cell clustering was performed to identify distinct cell populations, and cell types were annotated based on canonical marker genes using the SingleR algorithm and manual validation against known cell type signatures. To investigate the intercellular communication dynamics between immune cells and RNA-modified tumor cells, we employed the CellChat R package. This analysis was performed to identify and visualize the ligand-receptor interactions among different cell population.

### Identification and analysis of prognostic RMG

To identify RMGs with significant prognostic value for PFS, we employed the least absolute shrinkage and selection operator (LASSO) Cox regression model. Differentially expressed genes (DEGs) from sequencing data of tumor samples in the in-house cohort were visualized using the R package “ggplot2”. DEGs were selected based on a fold-change >1.3 and a p-value < 0.05. The mutational landscape of these RMGs and signatures from TCGA genomic data were analyzed using the “maftools” package.

### Predictive power assessment of IGF2BP1 and identified classification pattern

To evaluate the predictive power of IGF2BP1 and identified classification pattern, receiver operating characteristic (ROC) curves for 3-, 4-, and 5-year survival were plotted using the “timeROC” package in the internal cohort. High- and low-IGF2BP1 groups were stratified based on the optimal cut-off value determined by the “survival” package. Survival curves were compared using Kaplan-Meier analysis and log-rank test. Univariate Cox regression models determined the prognostic value of the IGF2BP1 expression.

### Chemotherapy sensitivity assessment

The NCI-60 is a well-characterized panel of 60 human cancer cell lines developed by the National Cancer Institute (NCI, https://dtp.cancer.gov/discovery_development/nci-60/cell_list.htm). This panel includes cell lines derived from nine different types of cancer: leukemia, melanoma, and cancers of the lung, colon, brain, ovary, breast, prostate, and kidney. The NCI-60 panel is widely used for drug discovery and cancer research because it provides a comprehensive representation of cancer diversity. By analyzing the NCI-60 tumor cell line panel, we explored the involvement of IGF2BP1 in drug sensitivity. Drug sensitivity data, quantified by half-maximal inhibitory concentration (IC50) values, were retrieved from the CellMiner database (https://discover.nci.nih.gov/cellminer/), a publicly accessible resource that integrates data on the molecular profiles and drug responses of the NCI-60 cell lines. Further analysis involved 218 FDA approved drugs and 574 drugs or compounds from clinical trials. The R packages “impute” and “limma” were utilized to evaluate the impact of IGF2BP1 on drug sensitivity. The impute knn function was employed to estimate missing data for certain medications.

### Immune cell type fractions analysis

The TIMER, CIBERSORT, and MCP-counter algorithms were utilized to calculate the infiltration levels of various immune cell types residing within each HNSC sample. These immune cell type fractions analyses employ deconvolution algorithms to test the presence of immune cells and their percentage. The ESTIMATE algorithm infers tumor cellularity and purity from transcriptional profiles. Using ESTIMATE, we calculated immune scores to assess infiltrating immune cells, finding that higher immune cell infiltration correlates with higher immune scores.

### Quantification of immune response predictors using IPS, and TIDE

The Immunophenoscore (IPS) predicts response to anti-CTLA-4 and anti-PD-1 therapies by measuring tumor immune proximity and intratumoral immune profile (21). This study assesses differences in CTLA-4-negative and PD-1-negative percentages across different subgroups. The Tumor Immune Dysfunction and Exclusion (TIDE) algorithm, which mimics the mechanisms of tumor immune evasion, predicts response to immunotherapy (22); higher TIDE scores indicate more severe immune evasion and lower response rates to immune checkpoint inhibitors.

### Consensus clustering

Consensus clustering was performed using the “ConsensusClusterPlus” tool in R to identify molecular subtypes. The optimal number of clusters (k) was evaluated by evaluating values between 2 and 10, with the clustering process repeated 1000 times to ensure reproducibility and robustness.

### Gene set variation analysis

Gene Set Variation Analysis (GSVA) was conducted on HNSC samples using the “GSVA” package in R. Enrichment scores, representing gene set activity, were calculated from transcriptome data. The Kyoto Encyclopedia of Genes and Genomes (KEGG) gene sets were utilized to determine the variations in functional signatures across samples.

### Statistical analysis

Statistical analyses were performed using GraphPad Prism 8.4.1. Student’s t-test or Wilcoxon rank sum test was used for continuous variables and chi-square test for categorical variables. For more than two groups, the Kruskal-Wallis test was used. *p < 0.05; **p < 0.01; ***p < 0.001; ****p < 0.0001.

## Results

### Identification of key prognostic genes IGF2BP1 in 59 RMGs and predictive ability

In total, 59 RMGs for RNA modifications (m1A, m6A, m6Am, m5C, m7G, ac4C, m3C, and Ψ) were obtained from a comprehensive review of previously published studies (**Supplementary Table S1**) (1, 2, 23–25). A chromosomal localization map of the 59 genes is displayed in **Supplementary Figure S1A**. The 59 aforementioned genes exhibited significant differential expression between tumor and normal tissues in the TCGA-HNSC dataset (**Supplementary Figure S1B**). We subsequently determined the prevalence of somatic mutations in 20 m6A and 16 m5C regulatory genes in HNSC. Among the 20 m6A regulators, WTAP and YTHDC2 exhibited the highest mutation frequency at 10.9%, followed by YTHDC1 at 9.4% (**Supplementary Figure S1C**). Within the 16 m5C regulators, TET1, DNMT3B, and DNMT3BA showed the highest mutation frequency at 13.8%, followed by TET3 at 12.1%, and NSUN2 and DNMT1 both at 10.3% (**Supplementary Figure S1D**). Further analysis of the 59 RMGs revealed a high prevalence of CNV mutations. RBM15B, ZC3H13, YTHDF2, and PUS3 exhibited widespread CNV amplifications. Conversely, KIAA1429, IGF2BP2, YTHDF3, DNMT3BA, NSUN2, NSUN3, TRMT10C, and NUDT16 predominantly showed CNV deletions (**Supplementary Figure S1E**).

**Figure 1:**
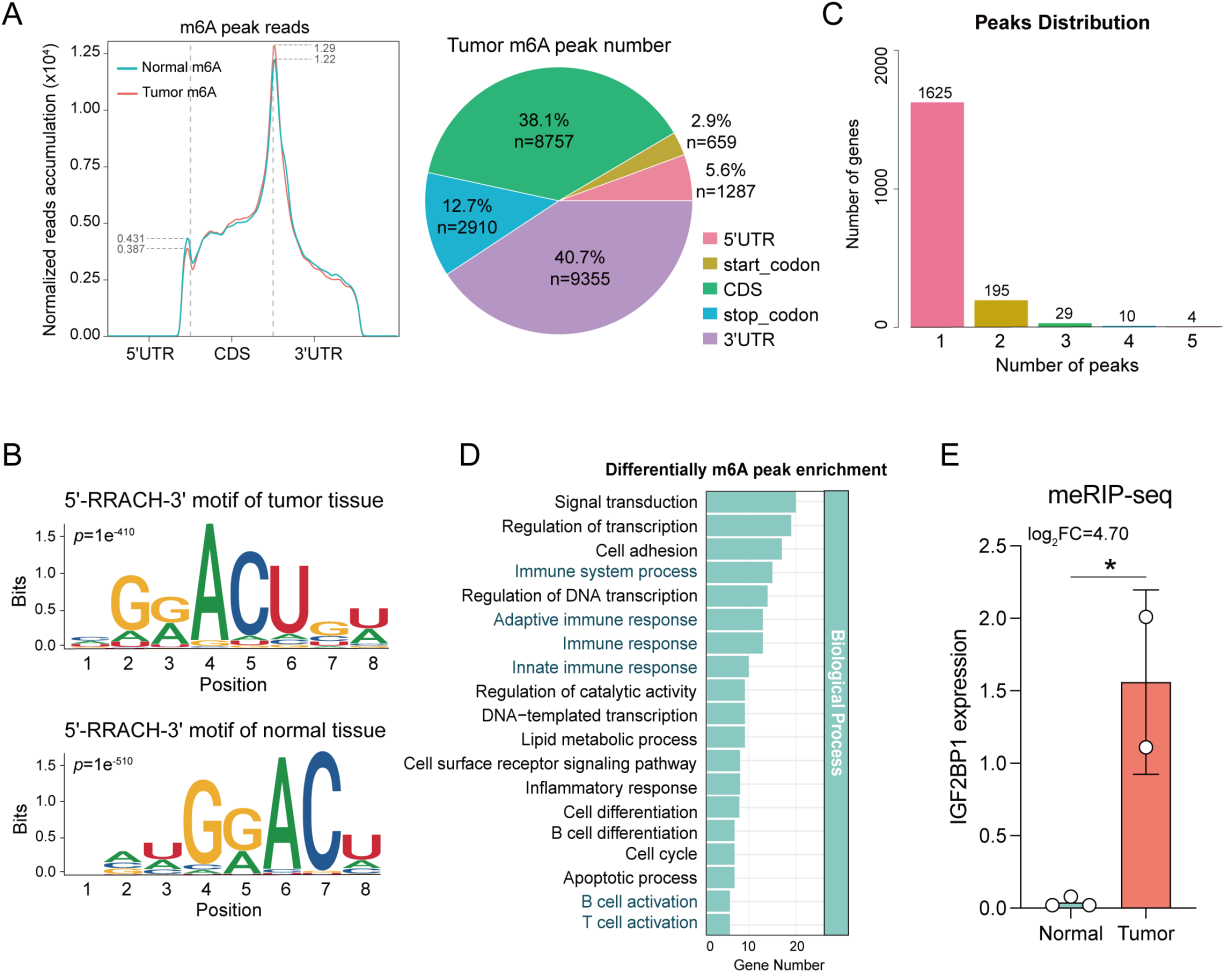
Comparative analysis of m6A levels between tumor and normal tissue. **(A)** The distribution of m6A peak reads and proportions in the 5’UTR, start codon, CDS, stop codon and 3’UTR in mRNA transcripts. **(B)** The m6A motif detected by MEME motif analysis. The RRACH (R=A/G, H=A/C/U) conserved sequence motifs for m6A-containing peak regions. **(C)** Genes with different number of peaks. **(D)** Enrichment analysis elucidates the functions of different m6A methylation modification genes. **(E)** IGF2BP1 exhibits differential expression in meRIP-seq analysis. *p < 0.05.

Given the ubiquity, abundance, and conservation of m6A as an endogenous modification in eukaryotic RNA, we conducted meRIP-seq analysis targeting m6A RNA methylation. Methylation of m6A in mammalian mRNAs predominantly occurs in the coding sequences (CDS) and 3’ untranslated regions (3’ UTR). Here, the m6A peaks were observed to be enriched in the CDS and 3’ UTR regions (**Figure 1A**), and only the consensus GGAC motif was detected (**Figure 1B**), indicating the successful enrichment of m6A-modified mRNAs. Considering that a gene may possess multiple m6A binding sites, we enumerated genes with varying numbers of peaks (**Figure 1C**). To elucidate the functions of genes with differential m6A methylation modifications, a Gene Ontology enrichment analysis was conducted, revealing that these genes were significantly enriched in various immune-related pathways, including Immune system process, Immune response, B cell activation, and T cell activation (**Figure 1D**). These findings suggest a potential correlation between m6A methylation modification and immune function.

**Figure 2:**
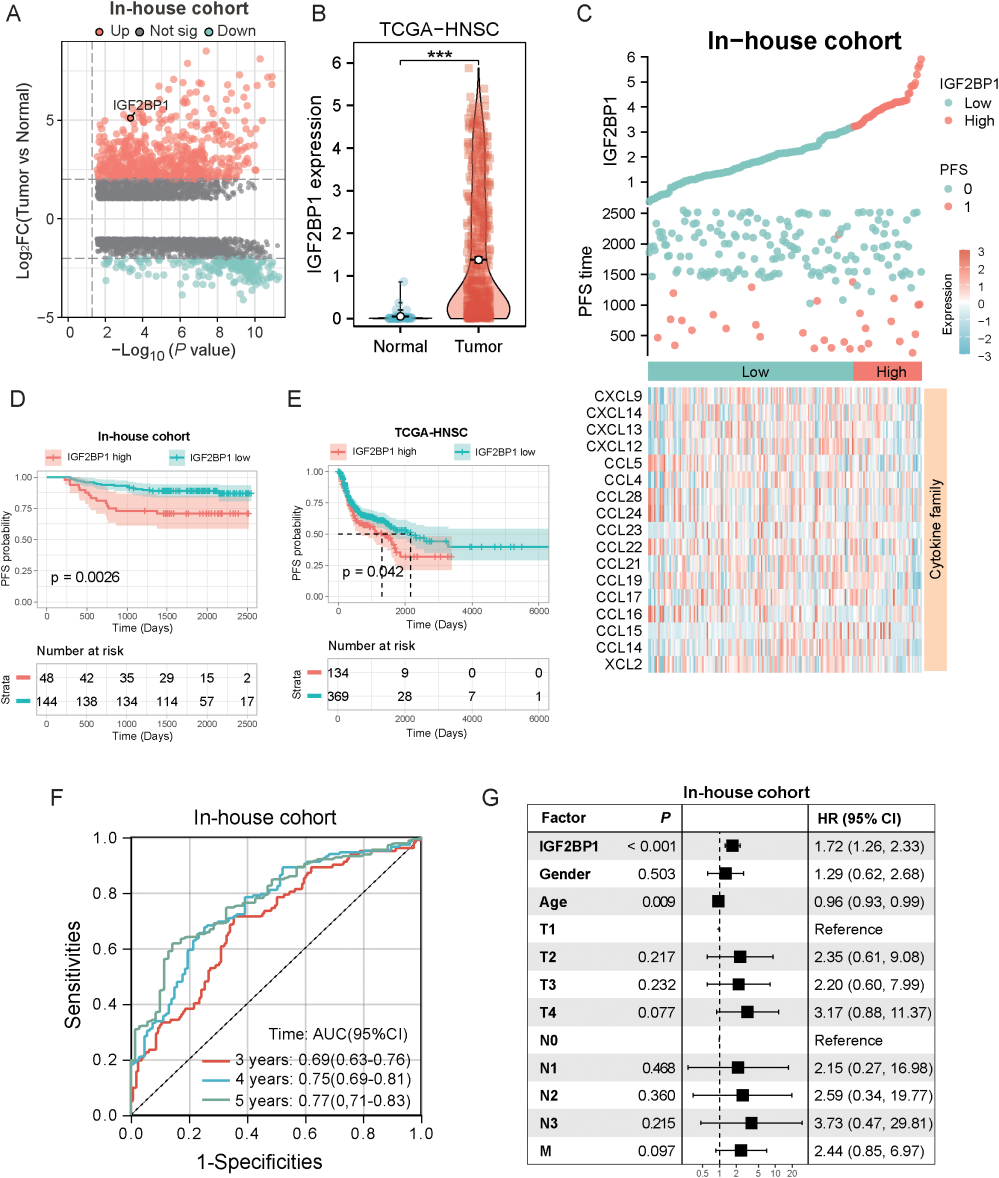
Screening of prognostically critical genes and the association with clinical features and prognostic predictive ability. **(A)** The volcano plot illustrates the differential gene expression between tumor and normal samples in the in-house cohort, highlighting the upregulation of IGF2BP1 in tumors (n=193). **(B)** The boxplot demonstrated that IGF2BP1 expression was significantly upregulated in the tumor samples of the TCGA-HNSC cohort. **(C)** Correlation of IGF2BP1 expression with that of the chemokine family. **(D, E)** In both the internal cohort **(D)** and the TCGA-HNSC cohort **(E)**, patients with high IGF2BP1 expression had shorter progression-free survival (PFS) and a worse prognosis. **(F)** ROC curve showing the predictive value of IGF2BP1 expression for 3-, 4-, and 5-year survival rates. **(G)** Univariate Cox analysis evaluate the prognostic value of IGF2BP1 expression in terms of PFS. ***p < 0.001.

To identify the genes most predictive of progression-free survival (PFS) in HNSC patients, we conducted LASSO regression and multivariate Cox regression analyses on 59 RMGs. This analysis identified IGF2BP1 as having the highest prognostic value for HNSC patients (**Supplementary Figures S2A, B**), and it also exhibited differential expression between tumor and normal tissues in meRIP-seq analysis (**Figure 1E**). In our in-house cohort, which comprised fresh tumor biopsy specimens obtained from 193 head and neck cancer patients at Fujian Cancer Hospital (from January 2015 to January 2018), a comparison of DEGs between cancer and paraneoplastic tissues revealed a significant upregulation of IGF2BP1 in cancer tissues (**Figure 2A**). Data from the TCGA-HNSC cohort supported this observation (**Figure 2B**). The best threshold value derived from the PFS analysis distinguished high- and low-IGF2BP1 expression levels. Chemokine families showed increased expression in the low-IGF2BP1 group in both the internal dataset and the external validation cohort. This upregulation was associated with a lower incidence of disease progression (**Figure 2C**; **Supplementary Figure S2C**). Patients in the high-IGF2BP1 group exhibited poorer tumor progression and worse PFS and OS prognosis (**Figures 2D, E**; **Supplementary Figure S2D**). The area under the ROC curve (AUC) demonstrated high predictive value, with scores of 0.69 at 3 years, 0.75 at 4 years, and 0.77 at 5 years (**Figure 2F**), and reached 0.930 in the TCGA-HNSC dataset (**Supplementary Figure S2E**). From the results of the univariate Cox analysis (**Figure 2G**), IGF2BP1 and age demonstrated strong survival predictive ability compared with other clinical features. Furthermore, IGF2BP1 expression was elevated in HPV-negative patients (**Supplementary Figure S2F**), those with lymphovascular invasion (**Supplementary Figure S2G**), and individuals with higher clinical T classifications (**Supplementary Figure S2H**). Additionally, IGF2BP1 expression exhibited a negative correlation with PD-L1 expression (**Supplementary Figure S2I**). Collectively, these findings suggest that high IGF2BP1 expression is associated with poorer prognosis and suboptimal treatment outcomes.

### High IGF2BP1 expression correlates with active cancer-related pathways and chemotherapy insensitivity

**Figure 3:**
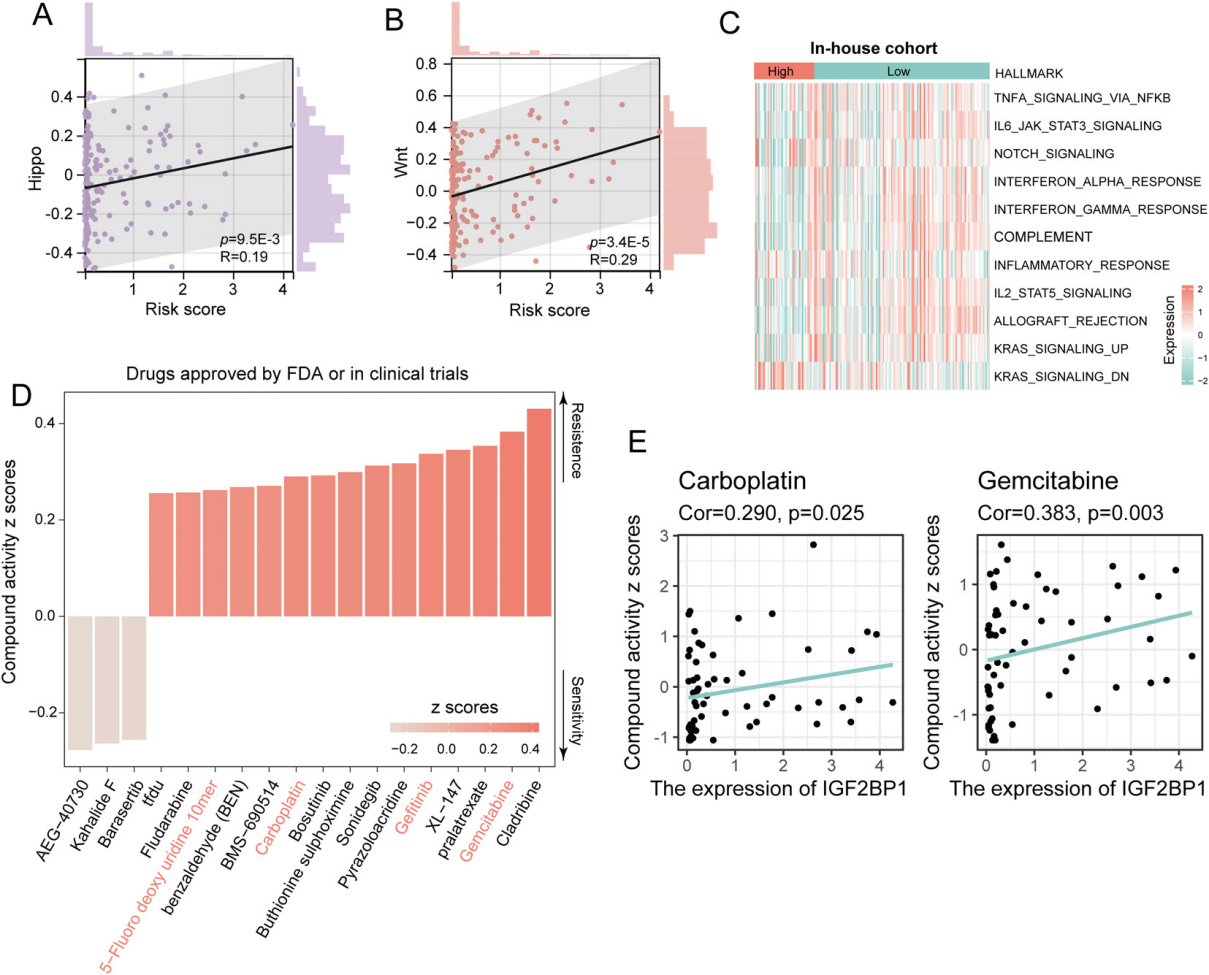
Assessment of the correlation between IGF2BP1 expression and cancer-related pathways, along with the prediction of chemotherapeutic drug sensitivity. **(A, B)** IGF2BP1 expression showed a positive correlation with the activity of cancer-related pathways. **(C)** Assessment of HALLMARK scores in patients with high and low IGF2BP1 expression. **(D)** The relationship between IGF2BP1 expression and chemotherapy drug sensitivity was evaluated. **(E)** The correlation between IGF2BP1 expression and the activity of carboplatin and gemcitabine compounds was assessed.

Next, to further elucidate the role of IGF2BP1 in cancer progression, we examined its association with cancer-related pathways and its impact on the sensitivity to common chemotherapeutic agents. We found that IGF2BP1 expression was positively correlated with scores in common cancer-related pathways, including the Hippo signaling pathway (**Figure 3A**) and the Wnt signaling pathway (**Figure 3B**). In our cohort, low levels of IGF2BP1 expression were associated with a high enrichment of cytokine-related HALLMARK pathways, such as complement signaling, IL2_STAT5_signaling, and inflammatory response signaling (**Figure 3C**). Additionally, chemotherapeutic agents used to treat HNSC, including 5-fluorodeoxyuridine, carboplatin, gefitinib, and gemcitabine, face resistance issues in patients with high IGF2BP1 expression, rendering these treatments less effective (**Figures 3D, E**).

### Evaluation of the TME and immunotherapeutic response

**Figure 4:**
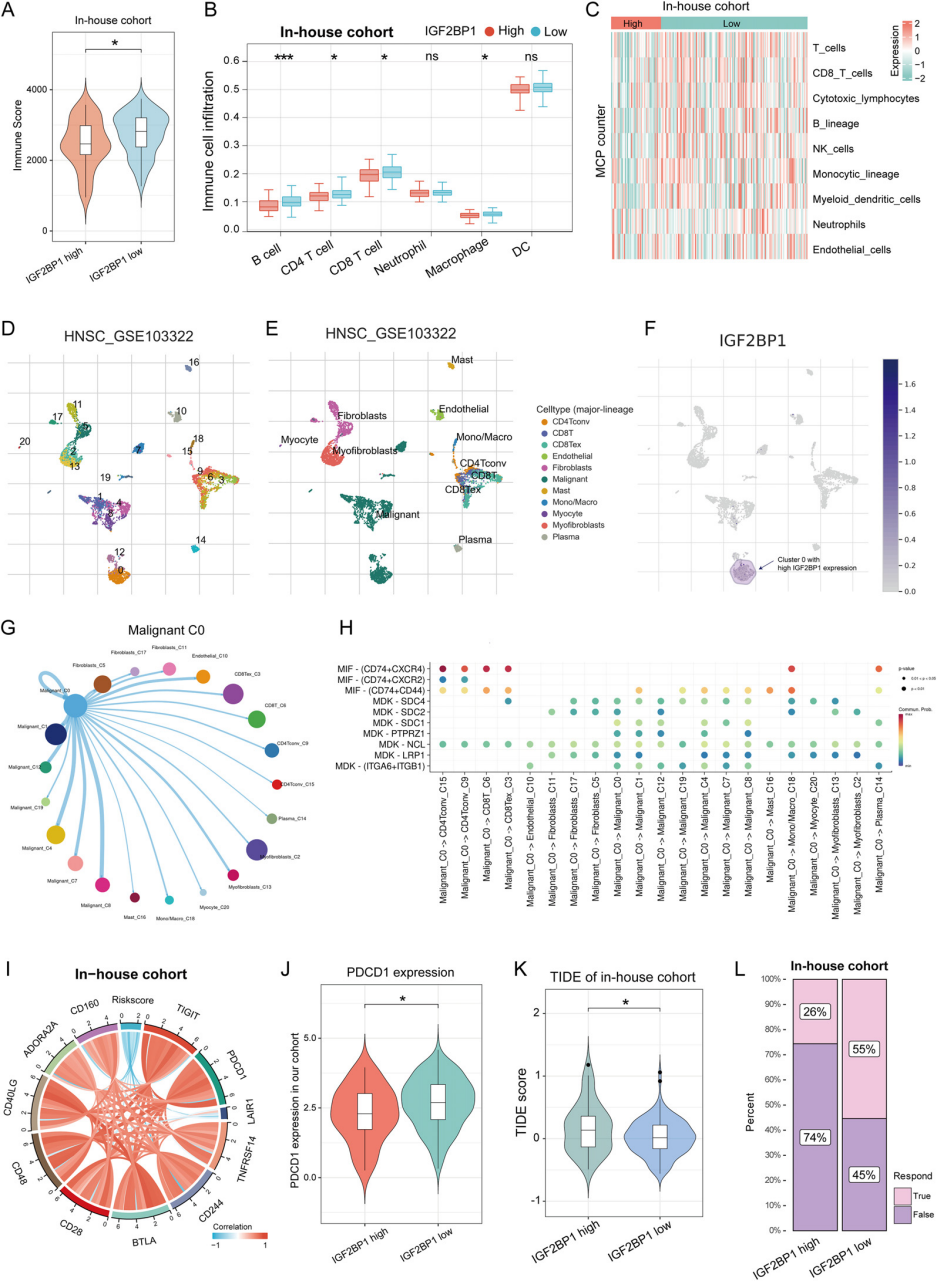
IGF2BP1 expression patterns correlate with the immune microenvironment and predict immunotherapy response. **(A)** immune score significantly differed between high and low IGF2BP1 expression subgroups. **(B, C)** Significant differences in immune cell infiltration were observed between high and low IGF2BP1 subgroups using TIMER **(B)** and MCP counter **(C)** algorithms. **(D, E)** Standard single-cell analysis process of HNSC samples, including dimensionality reduction **(D)** and annotation of cell types **(E)**. **(F)** Expression levels of IGF2BP1 across various cell subpopulations. **(G)** A circular plot illustrating the intensity of intercellular communication between malignant cluster 0 and other subpopulations, where line thickness indicates the strength of communication and the size of the bubbles reflects the number of interactions. **(H)** A bubble plot demonstrating the activity of ligand-receptor pairs during communication between malignant cluster 0 and other subpopulations. **(I)** IGF2BP1 expression correlated with immune checkpoint expression. **(J)** PDCD1 expression varied between high and low IGF2BP1 subgroups. **(K)** High IGF2BP1 expression was associated with higher TIDE scores. **(L)** More patients with low IGF2BP1 expression responded to immunotherapy. *p < 0.05; ***p < 0.001.

Given the insensitivity to chemotherapy, we shifted our focus to the immunotherapy response in patients with high IGF2BP1 expression. We analyzed immune microenvironment differences between high- and low-IGF2BP1 expression groups, assessing immune scores and cell infiltration. The low-IGF2BP1 group had higher immune scores (**Figure 4A**). The immune cell occupancy of each sample in the HNSC-TCGA cohort is illustrated in **Supplementary Figure S3A**, providing a visual representation of the infiltration of various immune cell types within each sample. From a quantitative point of view, TIMER algorithm revealed significant differences in six immune cell types between groups (**Figure 4B**), with T cells, CD8+ cells, B cells, and NK cells more active in the low IGF2BP1 group (**Figure 4C**). At the single-cell level, IGF2BP1 was predominantly expressed in malignant cells (cluster 0), with negligible expression observed in other cell types (**Figures 4D–F**); consequently, we designated cluster 0 as RNA-modified tumor cells. Notably, the immune cell type exhibiting the most significant interaction was CD8 Tex cells (**Figure 4G**), with the most active ligand-receptor pair identified as MIF - (CD74+CXCR4). The composition of the immune microenvironment critically modulates the efficacy of immunotherapy. We found that IGF2BP1 expression was negatively correlated with immune checkpoint expression validated in our in-house cohort (**Figure 4I**). Notably, PDCD1 showed significant differences between the high- and low- IGF2BP1 subgroups (**Figure 4J**). The low-IGF2BP1 group had a lower TIDE score, indicating a stronger immune response (**Figure 4K**). Patients in the low-IGF2BP1 group exhibited higher immune responses in the HNSC patient cohort at Fujian Cancer Hospital (**Figure 4L**).

### IGF2BP1 promotes malignant biological behavior of HNSC cells

**Figure 5:**
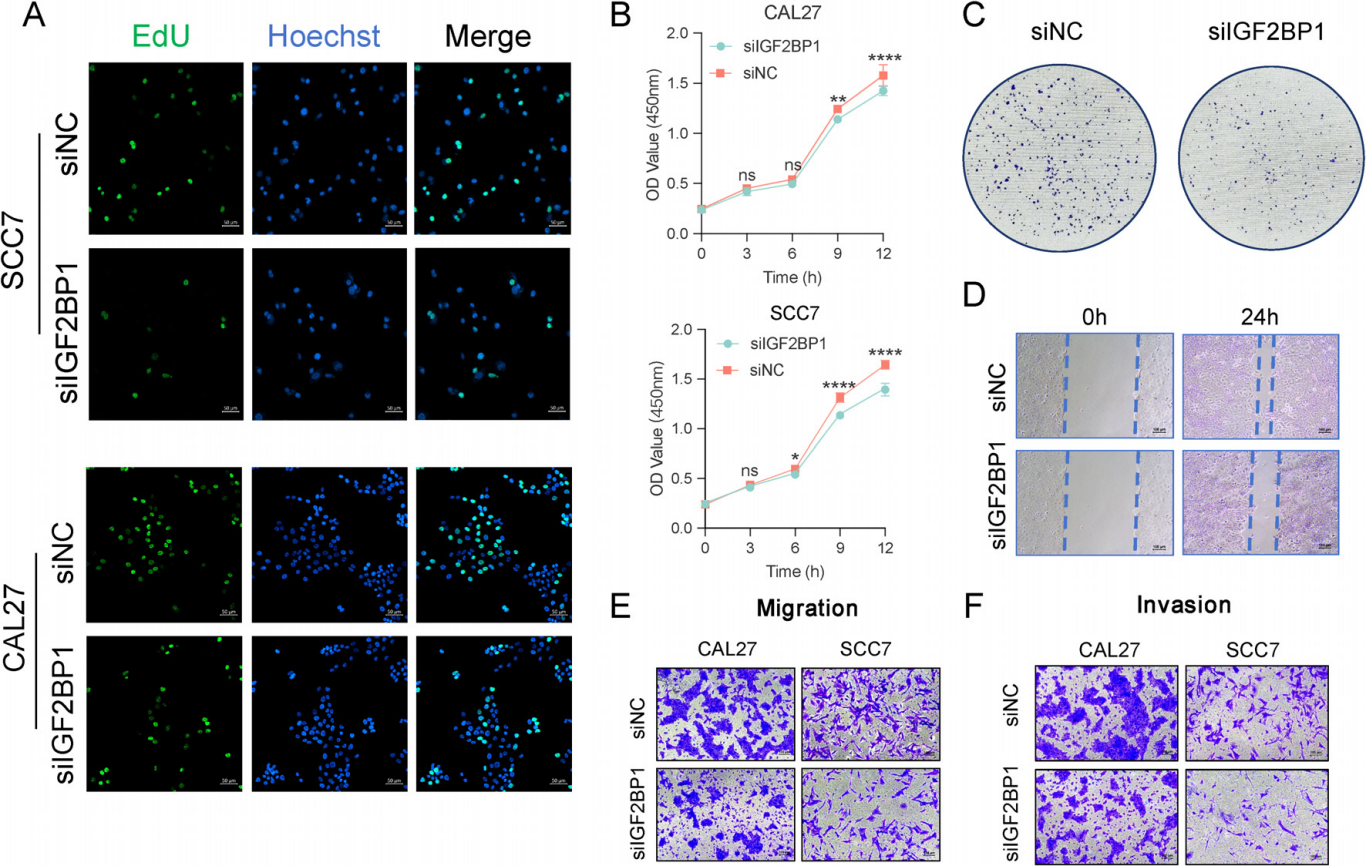
The malignant behavior related to IGF2BP1 expression. **(A)** The EdU assay assessed the proliferative capacity of cells; scale bar: 50 µm. **(B)** The Cell Counting Kit 8 experiment assessed overall proliferative capacity. **(C)** A clone formation assay was performed to assess the impact of altered IGF2BP1 expression on the self-cloning ability of cells. **(D)** A scratch healing assay was conducted to evaluate the impact of altered IGF2BP1 expression on cell migration ability. **(E, F)** The Transwell assay was used to evaluate migration ability **(E)** of cells after 24 h and invasive capacity **(F)** after 48h. *p < 0.05; **p < 0.01; ****p < 0.0001.

To further validate the function of IGF2BP1 as an oncogene, we constructed IGF2BP1 knockdown cell lines in the human-derived CAL27 and murine-derived SCC7 cell lines. High IGF2BP1 expression enhanced the proliferative capacity of HNSC cells, with more cells in the proliferative phase (**Figures 5A, B**). Additionally, high IGF2BP1 levels correlated with increased self-cloning ability (**Figure 5C**). IGF2BP1 downregulation reduced both migratory (**Figures 5D, E**) and invasive abilities (**Figure 5F**). Overall, these results indicated that IGF2BP1 plays a significant part in promoting the malignant biological behavior of HNSC tumor cells.

### HNSC patients were clustered into two subtypes with distinct clinical characteristics

**Figure 6:**
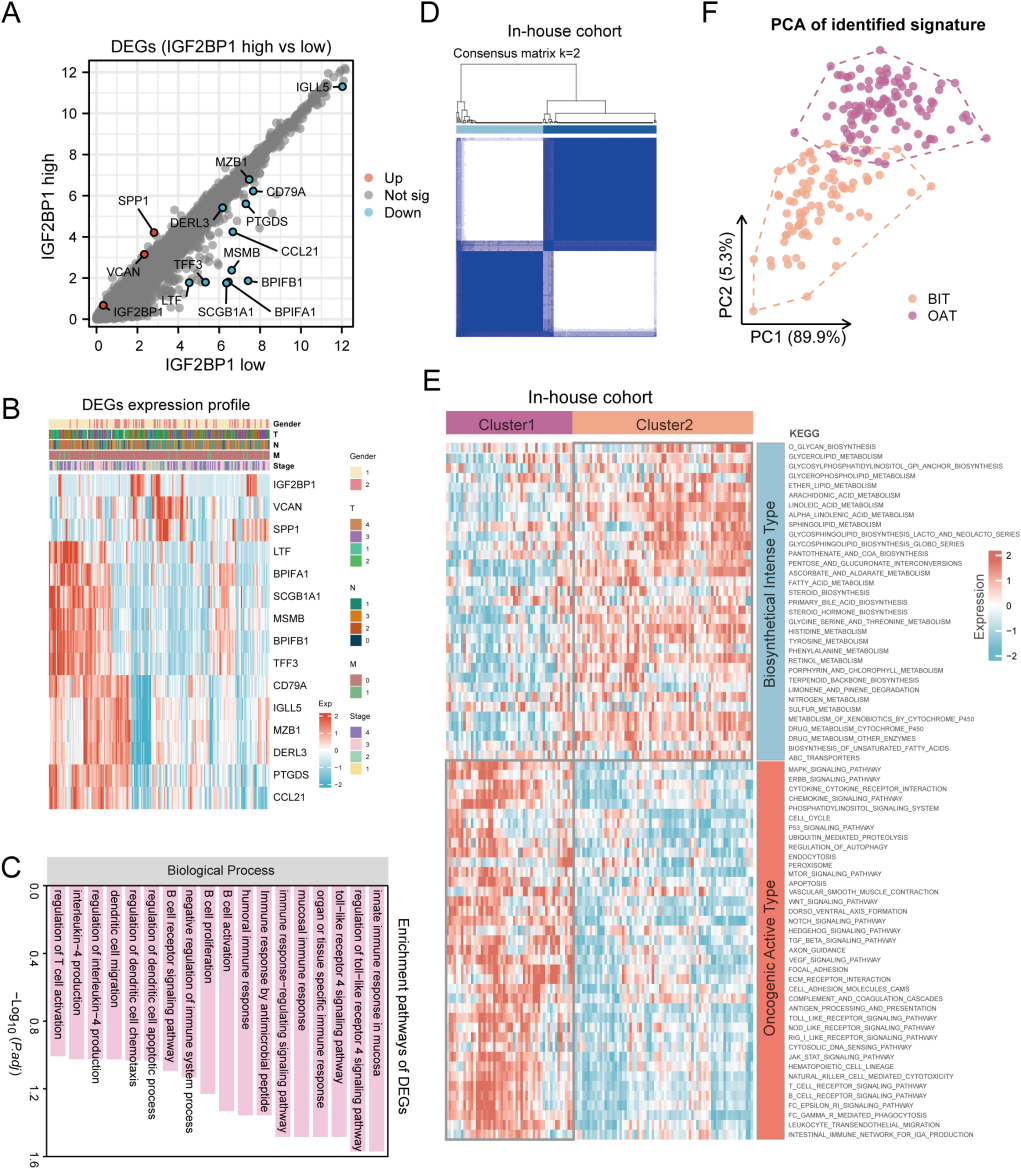
Consensus clustering identified two molecular subtypes in patients with HNSC. **(A)** Identification of DEGs between IGF2BP1 high- and low-expression groups in 193 HNSC samples; **(B)** Expression patterns of these DEGs; **(C)** Enriched pathways associated with identified DEGs; **(D)** Heatmap illustrating consensus clustering (k = 2) in 193 HNSC samples. **(E)** Heatmap depicting pathway scores for BIT and OAT molecular subtypes. **(F)** Principal component analysis plot demonstrating distinct expression patterns between BIT and OAT subtypes, with orange dots representing BIT and purple dots representing OAT.

Although HNSC patients were categorized into two groups based on PFS prognosis-related IGF2BP1 expression levels, the underlying genetic changes remain unknown. To address this, we investigated the potential transcriptional expression changes of IGF2BP1 alteration in RNA modification patterns. Using the limma method, we identified 15 DEGs associated with high- and low-IGF2BP1 expression, which are considered characteristic genes related to RNA modification (**Figure 6A**). The expression of these genes and their correlation with clinical features are shown in **Figure 6B**. These genes are enriched in several immune-related pathways, including immune response-regulating signaling, B cell activation, and T cell activation regulation (**Figure 6C**).

Precise and detailed clinical typing is essential for individualized treatment and the optimization of medical resources. To achieve this, we mapped the pathway characteristics of HNSC samples using the KEGG database. Through consensus clustering with the k-means technique, we identified two distinct clusters, each characterized by unique pathway activity patterns (**Figure 6D**). Specifically, cluster C1 actively participated in the biosynthetic pathways like glycerolipid metabolism, arachidonic metabolism, and fatty acid metabolism. In contrast, cluster C2 showed low metabolic pathway activity but high oncogenic activation, including MAPK signaling, ERBB signaling, cell cycle, mTOR signaling, and WNT signaling pathways. Thus, C1 was defined as the biosynthetical intense type (BIT), and C2 as the oncogenical active type (OAT) (**Figure 6E**). PCA revealed distinct transcriptional profiles and heterogeneity, showing strong separation between samples from the two clusters (**Figure 6F**). Similarly, in TCGA-HNSC samples, two distinct groups were identified based on the aforementioned clustering (**Supplementary Figures S4A, B**).

### The role of identified classification in clinical relevance and immunotherapeutic benefits

**Figure 7:**
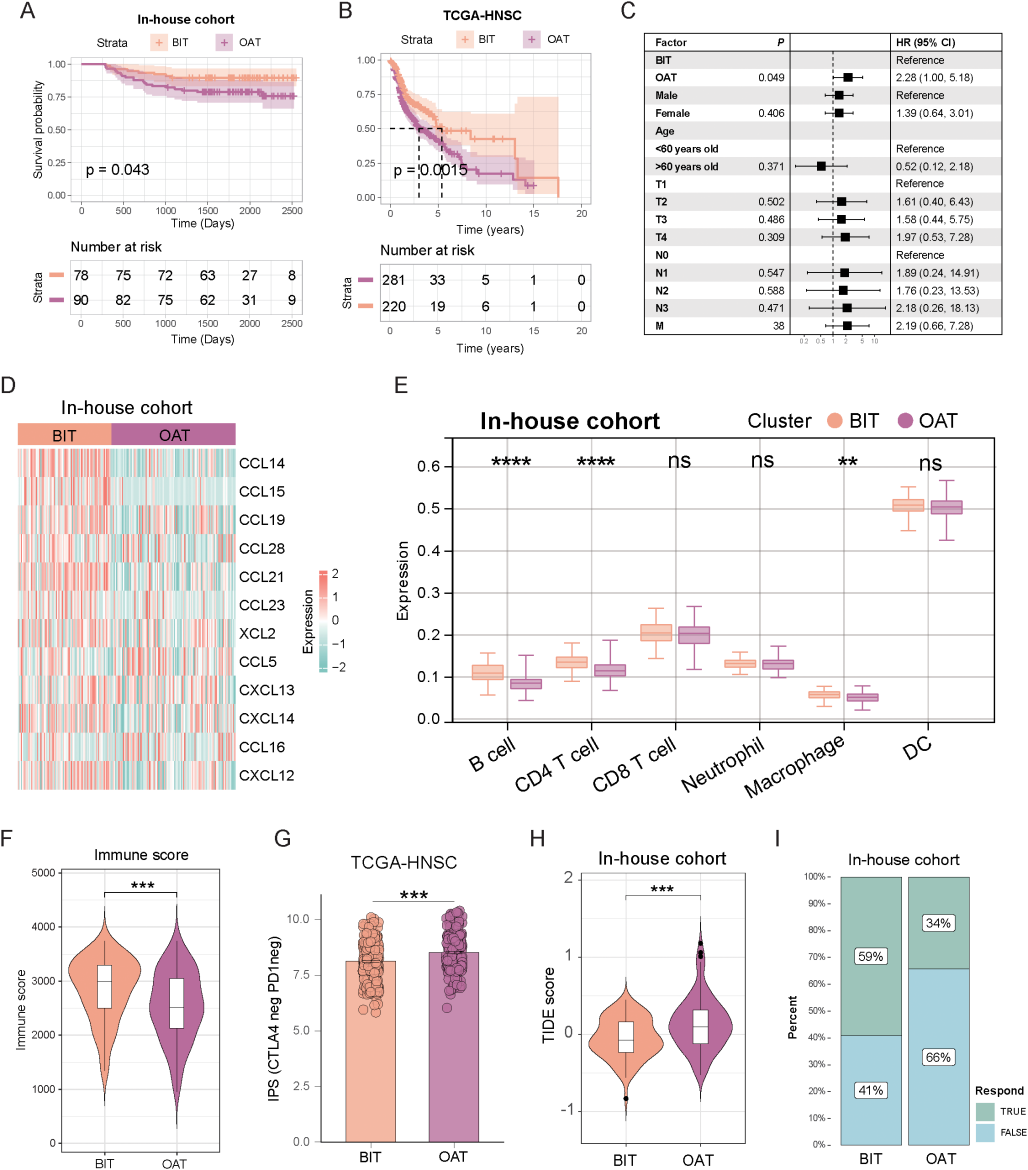
The two subtypes exhibited distinct prognostic outcomes, tumor microenvironment characteristics, and responses to immunotherapy. **(A, B)** Kaplan–Meier disease-free survival curve for all patients with HNSC assigned to BIT and OAT subtypes in the in-house cohort **(A)** and TCGA-HNSC cohort **(B)**. **(C)** Univariate Cox analysis evaluated the PFS prognostic value of our classification. **(D)** Heatmap displays the expression of chemokine families across the two subtypes. **(E)** Box plot illustrates the distribution of six immune cell populations scores between the subtypes. The upper, middle, and lower horizontal lines in the box represent the upper quartile, median, and lower quartile, respectively. **(F)** Violin plots highlight variations in immune scores between subtypes. **(G)** The IPS scoring system in the OAT subtype exhibits a higher percentage of CTLA-4 negative and PD1 negative. **(H)** TIDE scores of the two subtypes show significant differences. **(I)** A higher proportion of patients with the BIT subtype showed a positive response to immunotherapy. **p < 0.01; ***p < 0.001; ****p < 0.0001.

To evaluate the clinical application value of this classification, we assessed its prognostic significance and predictive efficacy for immunotherapy outcomes. In both the in-house cohort and TCGA-HNSC dataset, patients with BIT had longer progression-free survival and a significantly better prognosis (**Figures 7A, B**). Univariate Cox analysis indicated that patients in the OAT group had a hazard ratio of 2.28, predicting worse PFS (**Figure 7C**). Moreover, the chemokine family was significantly enriched in the BIT subtype, indicating more active cytokine chemotactic activity (**Figure 7D**). Consequently, we explored the infiltration of immune cells in the TME. In the in-house cohort, B cells and CD4+ T cells were significantly elevated in the BIT subtype (**Figure 7E**), consistent with findings from the TCGA-HNSC cohort (**Supplementary Figure S5A**). Overall, the BIT subtype exhibited higher levels of immune cell infiltration, suggesting a more active immune cytotoxic function (**Figure 7E**; **Supplementary Figure S5A, B**).

Given the observed immune cell infiltration patterns, it is unsurprising that the BIT subtype exhibited a higher immune score compared to the OAT subtype in the in-house cohort (**Figure 7F**). Moreover, the IPS score for CTLA4-neg PD1-neg in TCGA-HNSC was higher in the OAT subtype compared to the BIT subtype, suggesting that the OAT subtype has lower immune checkpoint expression and, consequently, a reduced likelihood of response to checkpoint inhibitor therapy (**Figure 7G**). Further analysis revealed that the OAT subtype had a significantly higher TIDE score than the BIT subtype in both the internal cohort (**Figure 7H**) and TCGA-HNSC (**Supplementary Figure S5C**), reinforcing the notion of a poorer immunotherapy response in the OAT subtype. Additionally, IFN-gamma expression was markedly lower in the OAT subtype compared to the BIT subtype (**Supplementary Figure S5D**). The response rate to immunotherapy decreased in the OAT subtype, as illustrated in **Figure 7I**. Collectively, these findings indicate that patients with the OAT subtype are less likely to derive benefit from immunotherapy.

## Discussion

In cancer biology, RMGs are pivotal due to their influence on tumor development (26). This study analyzed genetic variations among RMGs in HNSC, identifying prognostic genes like IGF2BP1, strongly linked to tumor progression. Elevated IGF2BP1 expression correlated with aggressive tumor behavior, chemotherapy resistance, and immune microenvironment alterations, indicating its central role in HNSC malignancy. We also introduced a new molecular classification, BIT and OAT, revealing unique clinical characteristics. BIT subtype exhibited better prognosis, heightened immune activity, and enhanced response to immunotherapy, promising for HNSC management.

RNA modifications and their regulatory mechanisms are closely intertwined with the TME in HNSC and other tumor types, impacting immune molecules, cells, and signal pathways (27, 28). Recent studies highlight the role of RNA modifications in regulating immune cell activation, infiltration, and subsequent immunotherapy outcomes, making them valuable targets for tumor immunotherapy (29–31). For instance, ALKBH5, an m6A demethylase, impacts T cell function and tumor growth (32). METTL3-mediated m6A modification influences NK cell homeostasis and function, affecting tumor growth and survival (33). Additionally, circIGF2BP3 overexpression in non-small cell lung cancer suppresses CD8+ T cell infiltration, compromising antitumor immunity (34). In this study, identification of key prognostic genes like IGF2BP1 enables its potential as a valuable biomarker, aiding in stratifying HNSC patients according to their risk of disease progression. The current study demonstrated that the MIF - (CD74+CXCR4) ligand-receptor pair is significantly active in tumor cells with elevated IGF2BP1 expression. MIF (Macrophage Migration Inhibitory Factor), a pivotal pro-inflammatory cytokine, orchestrates various immune responses and promotes the recruitment of immunosuppressive cells via its interaction with CD74 and CXCR4 receptors (35). This interaction is crucial for enhancing tumor immune evasion mechanisms within the TME (36). The activation of the MIF - (CD74+CXCR4) axis indicates that IGF2BP1-overexpressing tumor cells may facilitate immune evasion through this pathway, consequently undermining anti-tumor immune responses and adversely impacting patient prognosis in immunotherapy contexts.

HNSC exhibits diverse treatment responses and prognoses despite similar histologic types or TNM stages (37–39). The rapid advancements in precision medicine have significantly augmented our comprehension of tumor heterogeneity, offering deeper insights into the complex nature of cancer. Molecular subtyping of HNSC is advancing, with genomic studies identifying genetic alterations, including PI3KCA mutations, Kras activation, SMAD4 mutations, and activation of PI3K/Akt/mTOR and Wnt pathways (40–44). An HPV-related classification has been established, correlating subtypes with smoking behavior and tumor immune response, though immune cell components in the TME are overlooked (45). HNSC subtypes, like atypical, basal, classical, and mesenchymal, feature distinct characteristics, with the mesenchymal subtype displaying heightened epithelial-mesenchymal transition (EMT) and inferior survival outcomes, yet the unique role of immunotherapy in HNSC remains unexplored (46). Our study compiled 193 RNA expression profiles, categorizing samples into BIT or OAT subtypes based on pathway activity. The system demonstrated reproducibility, predictability, and substantial prognostic value, although internal cohort validation is warranted.

In recent years, the evaluation of immunotherapy efficacy and prognosis in specific tumor types has gained considerable attention in modern medical practice (47). Tumor-microenvironment interactions classify tumors into hot spots (abundant immune cells, responsive to immunotherapy) and cold spots (limited immune cells, less responsive) (48). Our study developed a predictive model for immune cell infiltration, also estimating chemotherapeutic drug sensitivity and immune checkpoint treatment response. Patients with high immune scores and immune cell infiltration, indicative of hot-spot tumors and robust immune responses, are likely to benefit from immunotherapy with improved prognosis.

While our study provides valuable insights, several limitations need to be acknowledged. The sample size, although substantial, may still limit the generalizability of our findings. Moreover, potential biases in the TCGA-HNSC dataset and our in-house cohort could influence the results. Further validation in larger, independent cohorts is necessary to confirm the prognostic value of the identified subtypes.

In conclusion, our study enhances comprehension of RNA modification regulators in HNSC by identifying key prognostic genes and elucidating the functional roles in cancer progression and treatment responses. We also introduce a novel HNSC classification based on transcriptomics, demonstrating significant predictive value for patient survival. These findings promise to advance personalized medicine in HNSC management through novel prognostic biomarkers and targeted therapies.

**Table 1:** Clinical features profile of in-house cohort patients.

Characteristics	Male	Female
N	136 (70.5%)	57 (29.5%)
Age, Mean ± SD	48.824 ± 10.827	47.421 ± 9.8488
T, n (%)		
1	24 (12.4%)	16 (8.3%)
2	31 (16.1%)	12 (6.2%)
3	44 (22.8%)	18 (9.3%)
4	37 (19.2%)	11 (5.7%)
N, n (%)		
0	13 (6.7%)	2 (1%)
1	45 (23.3%)	22 (11.4%)
2	53 (27.5%)	25 (13%)
3	25 (13%)	8 (4.1%)
M, n (%)		
0	128 (66.3%)	54 (28%)
1	8 (4.1%)	3 (1.6%)
stage, n (%)		
I	4 (2.1%)	0 (0%)
II	24 (12.4%)	15 (7.8%)
III	50 (25.9%)	23 (11.9%)
IV	58 (30.1%)	19 (9.8%)

## Data Availability

The data presented in this study are available in the GEO repository under accession number GSE103322 (https://www.ncbi.nlm.nih.gov/geo/query/acc.cgi?acc=GSE103322), as well as from TCGA-HNSC (https://portal.gdc.cancer.gov/projects/TCGA-HNSC), the NCI repository (https://dtp.cancer.gov/discovery_development/nci-60/cell_list.htm), and the CellMiner database (https://discover.nci.nih.gov/cellminer/). All original contributions discussed in this study are included in the article and its **Supplementary Material**. Additional information will be provided by the authors upon request, without any undue restrictions.
