# Supplementary material for: Tumor-related IGF2BP1-derived molecular subtypes to predict prognosis and immune microenvironment in head and neck squamous cell carcinoma
Source: Front Immunol. 2024 Oct 24;15:1469435. doi: 10.3389/fimmu.2024.1469435 (PMC11540706; doi:10.3389/fimmu.2024.1469435)
Supplement: Supplementary file 1 [file Presentation1.pdf]

Supplementary Materials for

Tumor-related IGF2BP1-derived molecular subtypes to predict prognosis and immune microenvironment in head and neck squamous cell carcinoma

Authors

Qin Ding[1,2#], Mingzhu Liu[1,2#], Yuhui Pan[1,2], Ziyi Wu[1,2], Jing Wang[1,2], Yi Li[1,2], Xiaoyong Liu[1,2], Jinghua Lai[1,2], Dan Hu[3*], Sufang Qiu[1,2**]

Affiliations

1. Department of Radiation Oncology, Clinical Oncology School of Fujian Medical University, Fujian Cancer Hospital (Fujian Branch of Fudan University Shanghai Cancer Center), Fuzhou 350014, China

2. Fujian Provincial Key Laboratory of Translational Cancer Medicine, Fuzhou 350014, China

3. Department of pathology, Clinical Oncology School of Fujian Medical University, Fujian Cancer Hospital (Fujian Branch of Fudan University Shanghai Cancer Center), Fuzhou 350014, China

Correspondence:

[*]Dan Hu: hudan@fjmu.edu.cn

[**]Sufang Qiu: sufangqiu@fjmu.edu.cn

[#] Contributed equally to this work.

Caption for supplementary material

Supplementary Experimental Procedures

Supplementary Table S1. Related genes in various RNA modification categories.

Supplementary Figure S1. (related to Fig. 1)

Supplementary Figure S2. (related to Fig. 2)

Supplementary Figure S3. (related to Fig. 4)

Supplementary Figure S4. (related to Fig. 6)

Supplementary Figure S5. (related to Fig. 7)

Supplementary Experimental Procedures

Total RNA isolation, construction, and sequencing of mRNA library

RNA extraction and preparation

Total RNA was extracted from tissues using TRIzol (Invitrogen, Carlsbad, California, USA) according to the manufacturer's instructions. Tissue samples were ground into powder under liquid nitrogen and transferred into a 2 mL EP tube containing 1.5 mL of TRIzol. The mixture was left to stand for 5 minutes. For cell samples, 1 mL of TRIzol was added per 1×10^6 cells, followed by vortex mixing and standing for 5 minutes. The mixture was then centrifuged at 4°C and 12,000xg for 5 minutes, and the supernatant was transferred to a new EP tube for extraction.

Chloroform/isoamyl alcohol extraction

300 μL of chloroform/isoamyl alcohol (24:1) was added to the supernatant and the mixture was vigorously vortexed. The mixture was centrifuged at 4°C and 12,000 × g for 8 minutes. The clear upper aqueous layer was carefully transferred to a new 1.5 mL EP tube. This process was repeated once. To the final upper aqueous layer, 2/3 of its volume of isopropanol was added (for low amounts of tissue or cells, 2 μL of 5 mg/mL glycogen can be added to aid precipitation). The mixture was gently inverted to mix and then placed in a -20°C freezer for 2 hours.

RNA precipitation and washing

The precipitation mixture was centrifuged at 17,500 × g and 4°C for 25 minutes. The supernatant was discarded, and the precipitation was washed with 0.9 mL of 75% ethanol. The precipitation was suspended by inverting the tube several times, then collected by centrifuging at 17,500xg for 3 minutes at 4°C. The supernatant was discarded, and the precipitation was dried in a biosafety cabinet for 3-5 minutes. Finally, 20-200 μL of DEPC-treated or RNase-free water was added to dissolve the RNA. The total RNA was then qualified and quantified using a Fragment Analyzer, Agilent 2100 Bioanalyzer (Agilent, CA, USA).

Library preparation

Library preparation was performed using the Optimal Dual-mode mRNA Library Prep Kit (BGI-Shenzhen, China). RNA was denatured at a suitable temperature to open the secondary structure, and mRNA was enriched using oligo(dT) attached magnetic beads. After a fixed reaction period at a suitable temperature, RNAs were fragmented with fragmentation reagents (BGI-Shenzhen, China).

First-strand cDNA was synthesized using random hexamer-primed reverse transcription, followed by second-strand cDNA synthesis. The double-strand cDNA was subjected to end-repair. An ‘A’ nucleotide was added to the 3’ ends of the blunt fragments through an A tailing reaction. Adaptors were then ligated to the cDNAs, and the library products were amplified through PCR and subjected to quality control.

Library cyclization and amplification

Single-stranded library products were produced via denaturation. The reaction system for circularization was set up to obtain single-stranded cyclized DNA products. Any uncyclized single-stranded linear DNA molecules were digested. The final single-strand circularized library was amplified using phi29 and rolling circle amplification (RCA) to produce DNA nanoballs (DNBs), which contained more than 300 copies of the initial single-stranded circularized library molecule. The DNBs were loaded into a patterned nanoarray, and PE 100/150 base reads were generated on the G400/T7/T10 platform (BGI-Shenzhen, China).

Data filtering

The raw data were filtered using SOAPnuke (v1.5.2) by:

1. Removing reads containing adapters (adapter contamination).

2. Removing reads with an unknown base ('N' base) ratio greater than 10%.

3. Removing reads with a low-quality base ratio (base quality $\leq$ 15) greater than 50%.

Clean reads were then obtained and stored in FASTQ format.

Reference genome and gene mapping

The clean data were mapped to the reference genome using HISAT (v2.1.0). Additionally, the clean data were mapped to assembled unique genes using Bowtie2 (v2.2.5). Gene expression levels were calculated using RSEM (v1.2.8).

Supplementary table

Table S1. Related genes in various RNA modification categories.

**Category**	**Gene Name**										
m6A Writers	KIAA1429	ZC3H13	METTL3	METTL14	WTAP	RBM15	RBM15B				
m6A Erasers	FTO	ALKBH5									
m6A Readers	YTHDC1	YTHDC2	YTHDF1	YTHDF2	HNRNPC	IGF2BP1	IGF2BP2	YTHDF3	IGF2BP3	HNRNPA2B1	RBMX
m5C Writers	TRDMT1	DNMT1	DNMT3A	DNMT3B	NSUN1	NSUN2	NSUN3	NSUN4	NSUN5	NSUN6	NSUN7
m5C Erasers	TET1	TET2	TET3								
m5C Readers	YBX1	ALYREF									
m1A Writers	TRMT6	TRMT61A	TRMT61B	TRMT10C	RRP8						
m1A Erasers	ALKBH1	ALKBH3									
m1A Readers	YTHDF1	YTHDF2	YTHDF3	YTHDC1							
ac4C Writers	NAT10	THUMPD1									
m3C Writers	METTL8										
m6Am Writers	PCIF1	METTL3	METTL4								
m6Am Erasers	FTO										
m7G Writers	RNMT	METTL1	WDR4								
m7G Erasers	NUDT16										
Ψ Writers	PUS1	PUS3	PUS4	PUS7	PUS9	TRUB1	TRUB2				

Supplementary figures

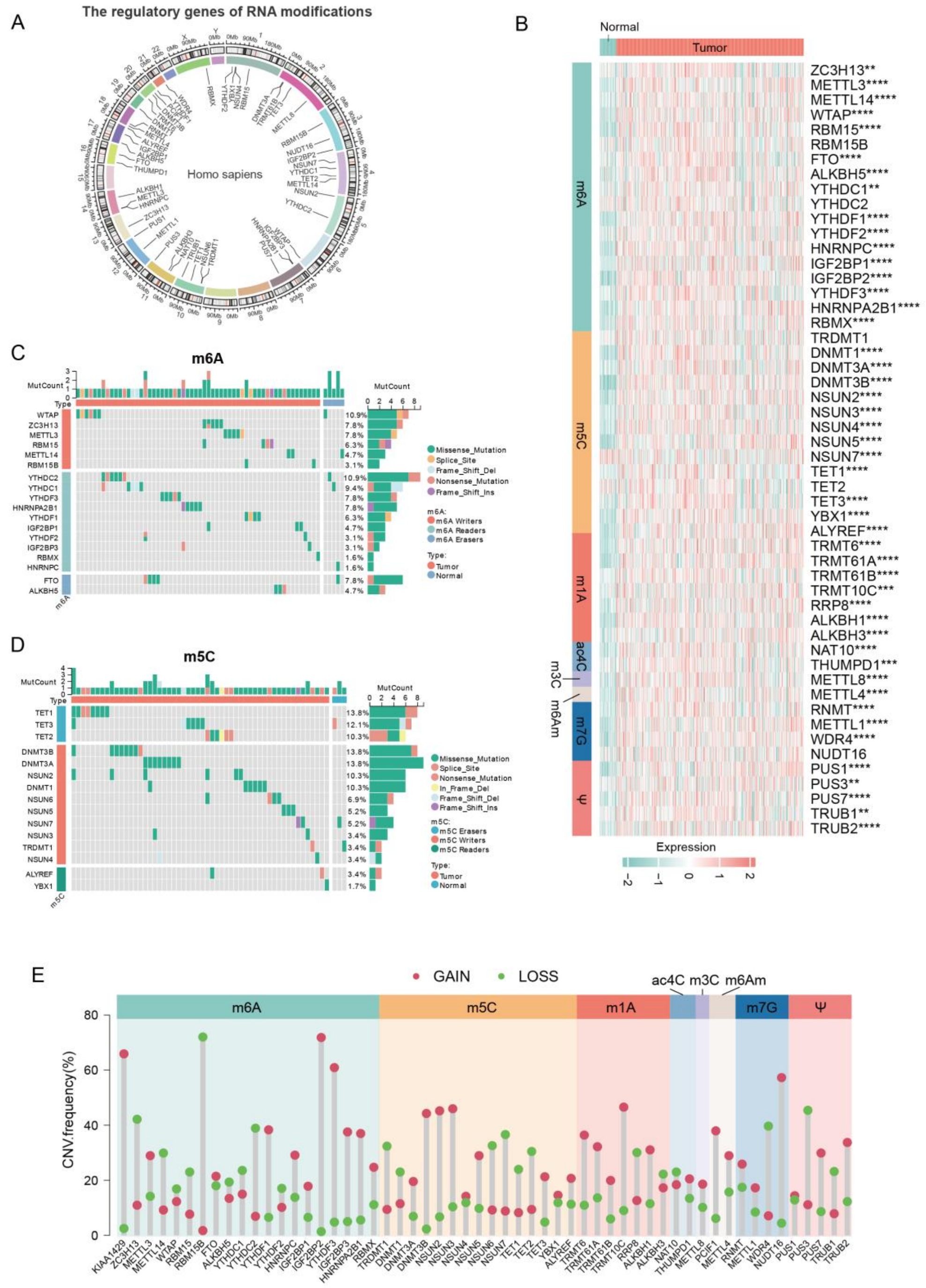

Figure S1. (A) Chromosomal localization of the 59 RMGs. (B) Differential expression of 59 RMGs in cancerous and normal tissues. (C–D) Genetic alterations of m6A regulators (C) and m5C (D) regulators, mostly including amplification, missense mutations, and splice site. The number on the right indicated the mutation frequency in each regulator. Each column represented individual patients. (E) The CNV

mutation frequency of 59 RMGs was prevalent. The column represented the alteration frequency. The amplification frequency, purple dot; The deletion frequency, green dot.

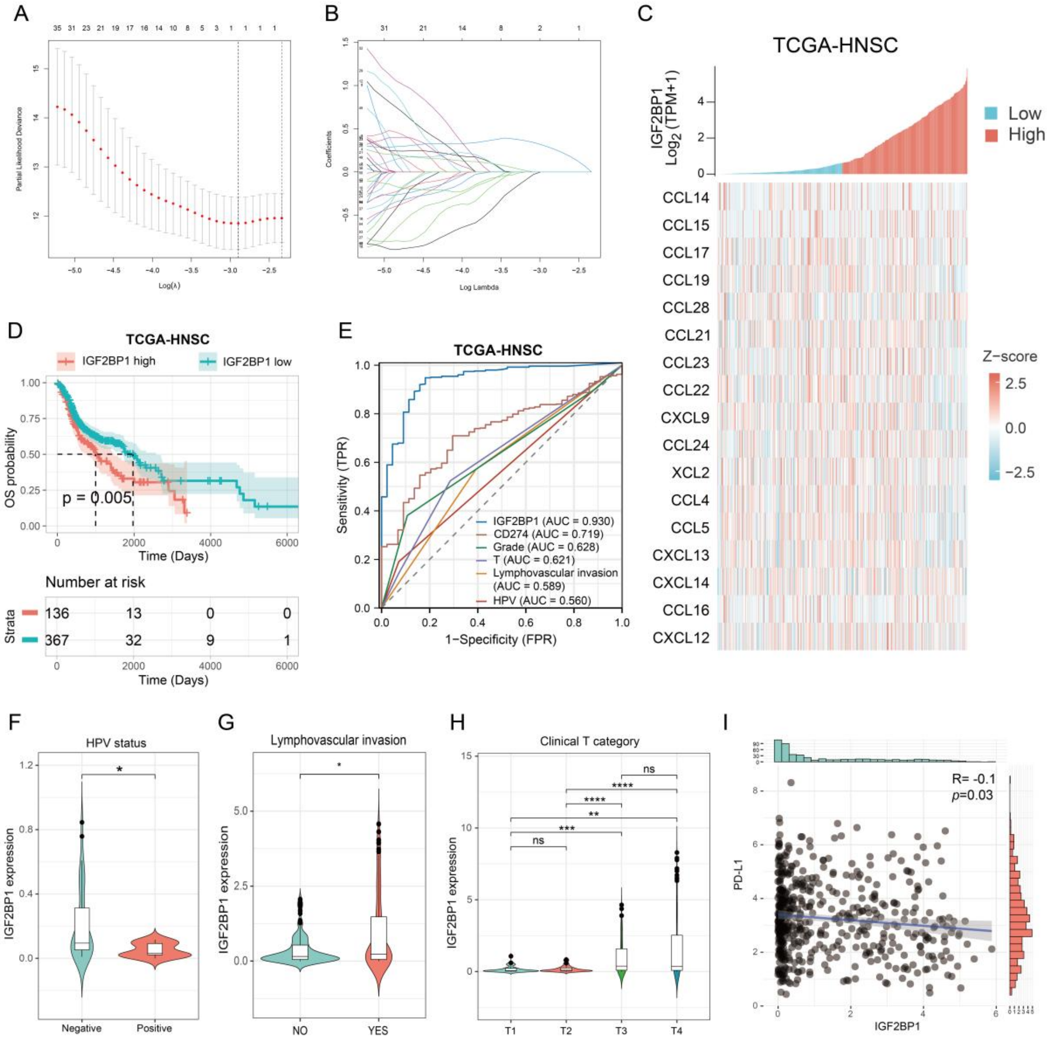

Figure S2. (A–B) Lasso regression identified IGF2BP1 as the gene with the highest weighted prognostic impact on HNSC among the 59 RMGs. (C) Correlation of IGF2BP1 expression with that of the chemokine family. (D) In the TCGA-HNSC cohort, patients with high IGF2BP1 expression had shorter overall survival (OS) and a worse prognosis. (E) IGF2BP1 expression demonstrates strong prognostic predictive ability. (F–H) Variations in IGF2BP1 expression according to HPV infection status (F), presence or absence of lymphovascular invasion (G), and clinical T category (H). (I) Correlation analysis between IGF2BP1 expression and PD-L1 expression.

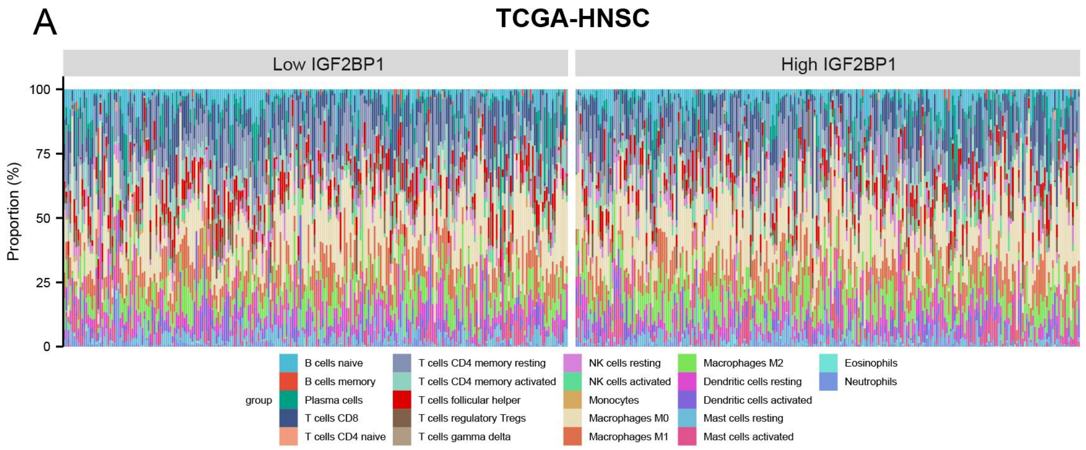

Figure S3. (A) The immune cell component of the infiltrate was evaluated for each patient sample in the IGF2BP1 high- and low-expression subgroups within the TCGA-HNSC cohort.

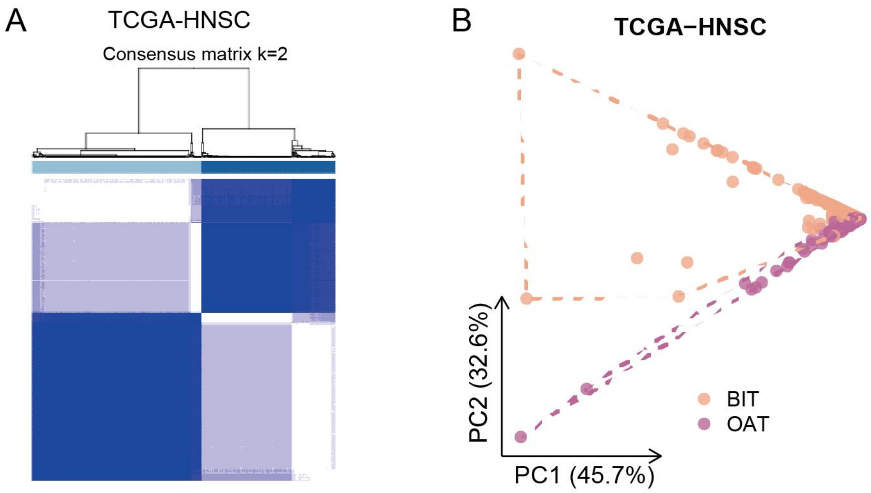

Figure S4. (A) Heatmap of consensus clustering solution (k = 2) in 504 HNSC samples. (B) Principal component analysis plots revealing distinct expression patterns among two subtypes; orange dots represent BIT subtype, and purple dots represent OAT subtype.

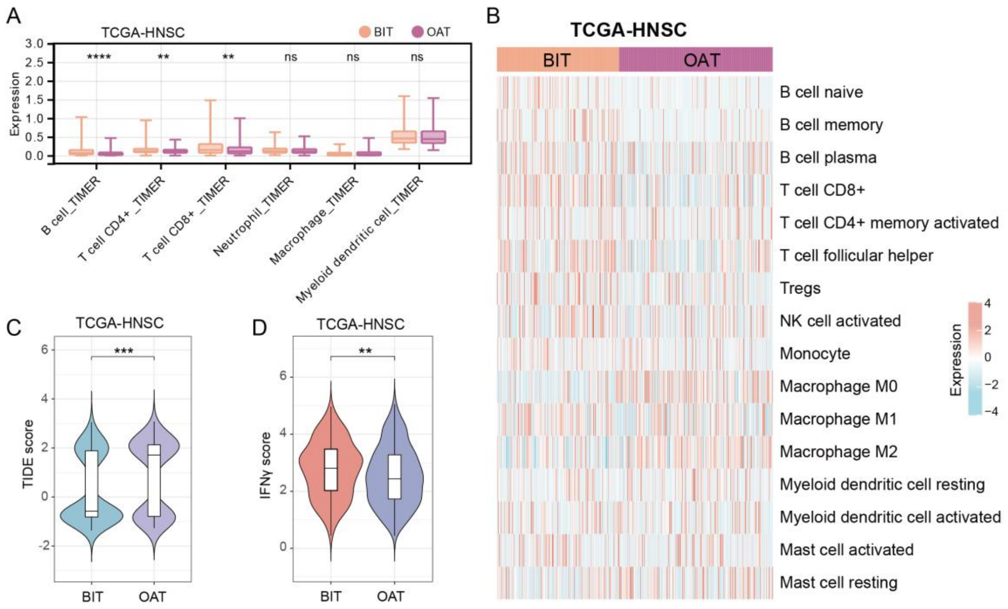

Figure S5. (A) Box plot of 6 immune cell population score among two subtypes. orange dots represent BIT subtype, and purple dots represent OAT subtype. The upper, middle, and lower horizontal lines of the box represent the upper, median, and lower quartile respectively. (B) Immune cell infiltration was assessed using the CIBERSORT algorithm for both the BIT and OAT fractions. (C) Differences in TIDE scores among patients with different subtypes in the TCGA-HNSC cohort. (D) Differences in IFNγ scores among patients with different subtypes in the TCGA-HNSC cohort. $^{**}p < 0.01$; $^{***}p < 0.001$; $^{****}p < 0.0001$.
